# Development of reading ability is facilitated by intensive exposure to a digital children's picture book

**DOI:** 10.3389/fpsyg.2014.00396

**Published:** 2014-05-02

**Authors:** Nobuo Masataka

**Affiliations:** Primate Research Institute, Kyoto UniversityInuyama, Japan

**Keywords:** digital book, literacy, language acquisition, reading

## Abstract

Here the author presents preliminary evidence supporting the possibility that the reading ability of 4-year-old children can be improved as a consequence of intensive exposure to the narrative in a digital picture book over a consecutive 5-day period. When creating the digital version used here, two additional functions were provided with it. First, the entire story was voice-recorded by a professional narrator and programmed so that it was played as narration from the speaker of an iPad. Next, as the narration of each digitized page proceeded, the character exactly corresponding to that pronounced by the narrator at that moment became highlighted in red. When the subjects' literacy capability with respect to the syllabic script of the Japanese language (kana) was evaluated before and after the exposure, their performance score was found to increase after the exposure to the digital book, whereas such a change was not recorded in children who experienced exposure to the printed version of the same picture book read to them by their mother. These effects were confirmed when the children were retested 4 weeks later. Although preliminary, the current study represents the first experimental evidence for a positive effect of exposure to digital books upon any aspect of child development.

## Introduction

Entertainment by looking at picture books and listening to their narration is enjoyed universally by young children and their caregivers (Aram and Aviram, 2009). Moreover, it is thought that through such opportunities, children experience a variety of emotions, acquire literacy skills as well as communication ability, and advance their intellectual thinking capability (Foorman and Torgesen, 2001; Masataka, 2003; McGee and Morrow, 2005; Arnold and Colburn, 2007). Our recent study showed that intensive exposure to narrative in children's books contributes to cultivating the capability of social perspective taking in schoolchildren (Tsunemi et al., 2014). In this regard, a recent dramatic change in the circumstances of publication in general, i.e., rapid dissemination of portable book readers and off-the-shelf digital books, is noteworthy because the distribution of children's books must unavoidably be affected by it.

There has been little consensus about the influence of the increasing prevalence of digital children's books upon the overall development of children's literacy abilities among specialists in childhood education and its related disciplines (Digital Book World, 2012; Trevian, 2012). Meanwhile, a recent survey of roughly 35,000 children in the UK found that the reading ability of those who read daily only on-screen was only about half as likely to be above average as that of those who read daily in print (Rideout et al., 2013). Those who read only on-screen were also three times less likely to enjoy reading very much. A more recent survey in the US found that the proportion of American teenagers who had read an e-book increased from 16 to 23% between 2012 and 2013, but that when it came to sharing books or reading with a child, most American adults who had read both print and e-books regarded print books as the better option (Pew Research Center, 2013). Given these findings, digital books appear somewhat detrimental to children. However, no detailed experimental research to examine this has been reported yet. The present study was performed to begin to address this issue.

Here the author compared the effects of intensive exposure to a picture book, either in a digital version (e-book) or a printed version (paper-book), upon the development of reading ability in 4-year-old Japanese boys. Before the commencement of the exposure, each child was tested as to his/her reading ability (referred to as the “Pre-test” below) so that all the participants were separated into two groups with equivalent average levels of reading skill. Thereafter, one of the groups (“Digital Book Group”) received exposure to the book in a digital version and the other received the same amount of exposure to a printed version (“Paper Book Group”) At the end of the exposure, the reading ability of all participants was tested again (“Post-test”) and the results were compared between the groups. As noted below, the participants in the Digital Book Group showed a significant increase in their performance on the Post-test as compared to the Pre-test. In order to confirm whether this increase was stable or not, the same testing was again conducted with the children 4 weeks after the Post-test. This is referred to as the “Follow-up Test” below. Since such an increase was not observed in the children of the Paper Book Group, this Post-test was not conducted with them.

## Method

This investigation was conducted according to the principles expressed in the Declaration of Helsinki. All experimental protocols were consistent with the Guide for Experimentation with Humans of the Primate Research Institute, Kyoto University, and were approved by the Institutional Ethics Committee of the Primate Research Institute, Kyoto University (#2011-150). The author obtained written informed consent from the parents of all participants involved in the study.

### Participants

The participants consisted of 30 4-year-old healthy children and their mothers. The author designed the present experiment to be conducted with a single gender participant group (male) partly because the main purpose of the study was to investigate the effects of exposure to digital or printed children's books upon the development of reading ability of the participants, not to investigate gender differences of these effects, and partly because the sample size was limited since this was a preliminary experiment.

All of the participants spoke Japanese as their first language. None of the participant children had been exposed to any language other than Japanese before the present study. The experimental room was a sound-attenuated playroom (3.5 × 5.5 m) familiar to all of the participants. Detailed information about the setting has already been documented elsewhere (Hayakawa et al., 2011). When they entered the room, each mother-child dyad was asked to sit side-by-side on a sofa. The mother was instructed to sit on the left side of the child, and to stay beside the child as long as they stayed in the room. Although the mothers were notified that reading of picture books to their children was of interest, they were not told specifically that effects of digital and printed versions of the books were to be analyzed.

### Material

**Figure 1:**
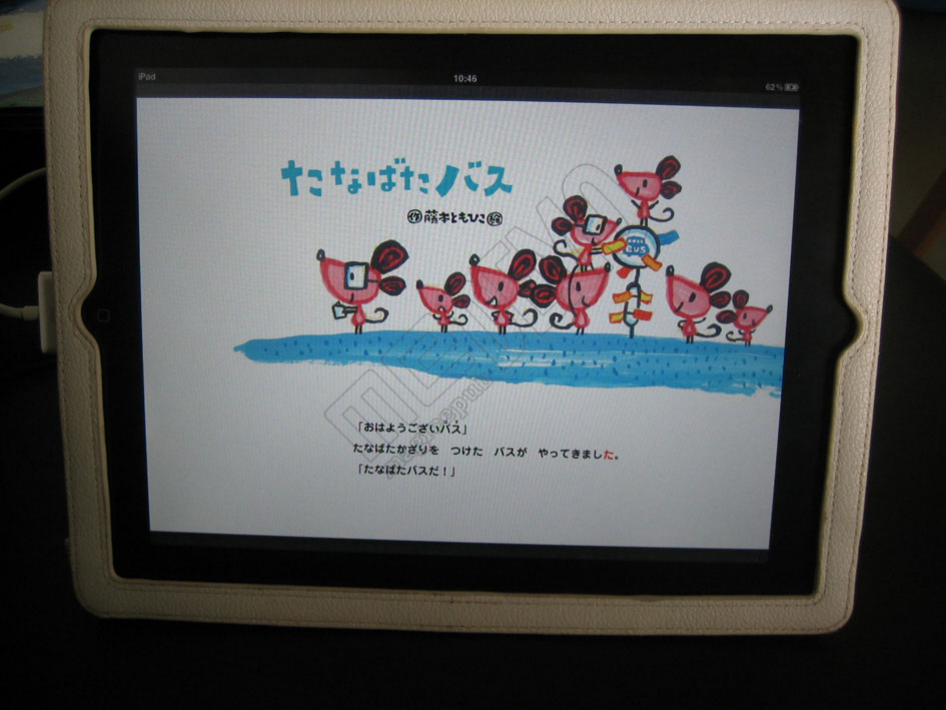
**Cover page of the digital version of “Tanabata Basu” presented on an iPad**. Note that a single character is presented in red color. At this moment, the narrator pronounces the syllable exactly corresponding to this kana character (ta), and this can be heard from the speaker of the iPad.

As the material used for the exposure, a 12-page-long Japanese picture book entitled “Tanabata basu,” which had been published for commercial use (Fujimoto, 2012) was adopted. The story described in this picture book consisted of a total of 140 sentences (576 characters, all of which were kana). For the printed version of the book to which the participants were exposed, a copy of that book was used as it was. For the digital version of the book to which the participants were exposed, original digitization of “Tanabata basu” was performed by scanning the entire book with the permission of its publisher and its author, so that it could be installed in an iPad. When creating the digital version, moreover, two additional functions were provided with it. First, the text of the entire story was voice-recorded by a professional narrator and programmed so that it was played as narration from the speaker of an iPad (narration function). In addition, each digitized page was elaborated so that as the narration proceeded, the character exactly corresponding to that pronounced by the narration at that moment became highlighted in red [all characters in the original book were printed in black ink (see Figure 1)]. Consequently, at the moment when one heard any particular syllable in the narration, one also saw a change from black to red of the color of the character that appeared on the iPad monitor (highlight function). When the participants of the Digital Book Group were exposed to the digital version of the picture book, both of these two functions were employed.

As the material used for testing the reading ability of the participants, a set of the 46 kana characters (Gothic in font, 50 DTP pt. in size, and black in color) was prepared.

### Procedure

On the day of the Pre-test as well as of the Post-test and of the Follow-up Test, a 38.1-cm screen personal computer was placed on the table of the experimental room in front of the child (at approximately 40-cm distance). An experimenter stayed in a room adjacent to the experimental room, observed the attention of the child, and started the stimulus presentation when the child was judged to attend sufficiently to the monitor. Once started, the entire set of 46 basic kana characters appeared consecutively with a 5-s interval between characters. As already noted, all of the syllables used in the Japanese language can be described by these characters, and all of these characters appeared in the children's book employed for exposure to the participants in the present study. The same computer was used for the stimulus presentation in the Pre-test, the Post-test, and the Follow-up Test.

Prior to the testing, the child was instructed to read the presented character aloud quickly if they could, whereas the mother was asked to keep silent, and the number of characters correctly answered during the interval after the presentation of each character was counted for each child by the experimenter. The order of presentation of the 46 characters was randomized.

On the days when the participant dyads were to be exposed to the picture book, they were asked to visit the laboratory. The parents were told to refrain from exposing their children to any book at home during the study period, and the experimental picture book was read the same number of times in both the Digital Book Group and the Paper Book Group.

Either the digital version or the printed version of the picture book was already lying on the table when the participant dyads entered the experimental room. For the dyads of the Digital Book Group, an iPad was prepared and the cover page of the book appeared on its screen. The mother was asked to take it, hold it with her hands between her right knee and the left knee of her child, and lightly touch the screen when she wanted to start to view the book. For the dyads of the Paper Book Group, the protocol was essentially identical with that for the dyads of the Digital Book Group, except that the mother of the dyad was required to read the story aloud until the end of the book as she normally read such books at home. During the reading, in both conditions, the mother was not allowed to make any additional comments about the book to her child. She was also asked to read it again after a 10-min break every day over the 5-day period.

## Results

Table 1 presents the results of comparisons of the mean number of characters the participants correctly answered between the Pre-test and Post-test in the Digital Book Group and the Paper Book Group. When these collected data were analyzed by a 2 (GROUP: the Digital Book Group vs. the Paper Book Group) × 2 (PHASE OF TESTING: Pre-test vs. Post-test) analysis of variance (by subjects ANOVA), the main effect for GROUP was not statistically significant [*F*_(1, 28)_ = 1.60, *p* = 0.22]. On the other hand, the main effect for PHASE OF TESTING was significant [*F*_(1, 28)_ = 34.80, *p* = 0.000002]. There was also a significant interaction between the two factors [*F*_(1, 28)_ = 20.83, *p* = 0.000091]. *Post-hoc* analyses using Tukey's tests revealed that the number of characters the participants of the Digital Book Group could read significantly increased in the Post-test as compared to the Pre-test (*p* = 0.00017). However, the number did not differ significantly between the Pre-test and Post-test for the participants of the Paper Book Group (*p* = 0.78). While 11 of the 15 children of the Digital Book Group learned 2–4 characters during the experiment, 12 of the 15 children of the Paper Book Group did not learn any new character.

**Table 1:** **Mean number of characters the participant children correctly answered (SDs) before (Pre-test) and after (Post-test) the exposure to the picture book**.

**Group**	**Pre-test**	**Post-test**
Digital Book	16.4 (4.9)	19.5 (5.7)
Paper Book	16.6 (5.3)	16.9 (6.3)

When the data were analyzed by another analysis of variance (by items ANOVA), the results were similar. While the main effect for GROUP was not significant [*F*_(1, 45)_ = 1.54, *p* = 0.27], that for PHASE OF TESTING was significant [*F*_(1, 45)_ = 29.96, *p* = 0.000003]. There was also a significant interaction between the two factors [*F*_(1, 45)_ = 18.20, *p* = 0.000061]. The number of participants who could correctly read a given character increased in the Post-test as compared to the Pre-test in the Digital Book Group (*p* = 0.00023) whereas this number did not differ significantly in the Paper Book Group (*p* = 0.82).

The mean number of characters the participants of the Digital Book Group correctly answered (SD) in the Follow-up Test was 19.9 (6.8), and that number did not differ significantly from that in the Post-test [*F*_(1, 14)_ = 0.71, *p* = 0.41].

## Discussion

The results of the present experiment revealed that the average number of kana characters each child could correctly read was increased between the Pre-test and Post-test only in the Digital Book Group. Individual variability in the learning appeared relatively small. Moreover, the stability of the change of the performance was also confirmed by the results of retesting that was performed 4 weeks later. These results clearly indicate that the digital picture book used here was more effective for literacy acquisition of the children than the printed version of the same book, though the sample size was limited as this was a preliminary study.

When interpreting these findings, it should be noted that the digital book used here was not one in which the printed text was simply digitized, but rather that additional functions were provided with the text, i.e., a narration function and a highlight function. In the present study, the effects of exposure to the simply digitized version of the text upon the development of reading ability in 4-year-olds were not investigated, but it seems highly probable that the additional functions must have contributed profoundly to the facilitation of literacy development in the Digital Book Group.

It is quite common for Japanese children to start to read some kana characters at the age of 4, when most of them enter kindergarten, and to be able to read and write all the characters in the kana script by the time they go to primary school (MEXT, Japan, 2013). Clearly, the facilitating effects of the functions introduced into the digital book in the present study upon the development of the reading ability of the children are expected to be specifically and closely related to the linguistic and educational circumstances in which Japanese children develop, and these findings cannot necessarily be generalized to children raised in other cultures in which alphabetical writing systems are used. For example, audio-visual association between each character and its corresponding syllable is possible through the functions added here when learning Japanese, but not when learning other languages such as English. It is also true that the narrator of the story was professional and the same for all the children in the Digital Book Group, whereas the presentation was dependent on the mother of each child and consequently not controlled in the Paper Book Group. The level of familiarity, as well as the variability, of the voices was not controlled, since the same unfamiliar voice was used for all the children in the Digital Book Group whereas the familiar voice of the mother was used for the children in the Paper Book Group.

Nonetheless, this hardly negates the implications of the present findings that the use of digital picture books could exert a positive influence upon child development. The present experiment is a first attempt to empirically examine the oft-stated criticism of the narration function provided with most digital children's books, namely, that children's books should be read aloud to children by caretakers (Digital Book World, 2012), and appears to provide evidence against that notion. The present findings suggest that digital books would not necessarily be detrimental. A digital device, no matter how highly elaborated, must be a tool, and as long as it remains a tool, how effectively it can be used depends upon the person who uses that tool. What is needed in order to evaluate the influence of the increasing prevalence of digital books upon child development is to expand our scientific knowledge, and as a first such attempt, the author has reported the present experimental evidence. Finally, it would also be of interest to examine the impact of e-reading on writing, because a number of studies (e.g., Longcamp et al., 2003, 2005) have shown that writing by hand vs. typing on a computer influences reading abilities. Studies have consistently shown that the experience of writing by hand gave rise to better letter recognition than the experience of typing. It would be of interest to determine whether the reverse is also true, an issue that will be investigated in the near future.

## Funding

This research was supported by a grant-in-aid (#25285201) as well as by the Grants for Excellent Graduate Schools program, from the Ministry of Education, Science, Sports and Culture, of the Japanese government.

### Conflict of interest statement

The author declares that the research was conducted in the absence of any commercial or financial relationships that could be construed as a potential conflict of interest.
